# Diabetes care in an acute psychiatric inpatient setting: a logic model for service delivery

**DOI:** 10.1192/bjo.2021.847

**Published:** 2021-06-18

**Authors:** Zoe Goff, Allan House, Elspeth Guthrie, Hannah Weston, Laura Mansbridge

**Affiliations:** 1 Leeds and York Partnership Foundation Trust, University of Leeds; 2 University of Leeds; 3 Leeds and York Partnership Foundation Trust

## Abstract

**Aims:**

To develop a logic model that illustrates the steps needed to develop an effective intervention for diabetes management in a psychiatric inpatient setting, as the point of admission to a psychiatric inpatient unit may present as an opportune time for improving diabetes care.

**Method:**

We undertook (i) a survey of diabetes care among inpatients in a Mental Health Trust in England, comparing care to the National Health Service (NHS) Core National Diabetes Standards (ii) interviews with key clinical staff to understand challenges in delivering good diabetes care (iii) a review of current UK guidance on standards for diabetes care. On the basis of the findings we developed an initial logic model for service delivery.

**Result:**

Among 163 inpatients reviewed, 44 (27%) had a diagnosis of diabetes, and only 3 (7%) had all three National Institute for Health and Care Excellence (NICE) treatment targets within range. Staff identified needs for regular training, better understanding of roles in shared care, and good quality IT support. We developed a logic model that illustrates the steps needed to develop an effective intervention for diabetes management in a psychiatric inpatient setting.

**Conclusion:**

Admission to a psychiatric inpatient setting provides an opportunity in which diabetes care may be optimised. The quality and understanding of diabetes care will need to be enhanced if this opportunity is to be exploited.

